# The Champions of Silence

**Published:** 1918-11-16

**Authors:** 


### THE NURSES' DAY AND FUTURE OUTLOOK.

### The Champions of Silence.

My congratulations to "F. N." on having replied to my queries instead of following the example of certain other correspondents whose arguments consist of shutting the door with a bang. "F. N." has chosen the better plan of pondering the subject for three weeks, and coming back with answers to all but one, the purport of which she does not understand. This is probably due to my clumsy way of putting the question, but it was of no importance. What interests me is that "F. N.'s" views of the nursing situation turn out to be practically identical. Whether this was so when she complained of my "baneful influence," or whether three weeks of pondering have converted her, does not appear. The fact remains.

In that letter "F. N." made very specific charges against me, and, lest they be forgotten, I desire to rehearse them. Here are some of them—ignorance of the tenets and ideals of the nursing profession; being after the manner of a Socialist agitator and an obvious outsider; ignorance of the meaning of the word "loyalty"; striving to promote a spirit of unrest among the rank and file. On the other hand I am accredited with a modicum of honesty, else presumably a gallows at Tyburn would have been my fate. It has refreshed and encouraged me to read the views held by "F. N.," who desires to relegate me to silence, and to an obscurity from which I should never have emerged. She regards the British public on the whole as utterly unacquainted with the important and responsible duties which nurses have to discharge, and which they think consists mainly of bed-making. She also holds that the hospital matron is helpless with regard to her pay and conditions of employment, being confronted with the alternative of falling in with existing circumstances, or being requested by the committee to resign. Up to this point we travel together, but here the road bifurcates. "F. N." considers that the only becoming course for the victims to pursue is to suffer in silence, and that any agitation on their part for betterment amounts to disloyalty to "the ideals and tenets" of the profession. I, on the other hand, am fully convinced that they have tried the experiment of suffering in silence long enough. It is sixty years since Miss Nightingale complained of the shortage of nurses owing to inadequate remuneration, and if the profession follow the advice of "F. N." they will remain as bondslaves for sixty more. This is not a speculative estimate. It must be so. Suppose for the sake of argument that "outsiders" and "Socialist agitators" take up the championship of the nurse, the answer is immediately made that she is perfectly satisfied with her conditions of service. Has "F. N." failed to see the import of Major Chapple's correspondence? Major Chapple is a medical man and a public man, and he has taken much interest in nursing affairs. He finds that in the biggest public hospital in the kingdom the earnings of nurses are being spent by the committee in charity, and when he challenges the ethics and the justice of this he is assailed not only by the chairman for not knowing what he is talking about, but members of the "London" staff write to the press declaring that they are perfectly satisfied and in the lap of luxury. I venture to suggest that this incident is a perfect answer to "F. N.," and to all such whose attitude is that the rank and file of the profession should remain inarticulate. The nurse, from a sense of dignity, is to bear her burden without complaint, and the outsider is to mind his own business!

There is no room for doubt that the silence of the nurse is the instrument of her destruction. The world, unhappily, is not fairyland, and no knight in shining armour is likely to appear in the board-room to challenge the recreant committee to mortal combat, and hence I cannot regard "F. N.'s" as a business or a practical proposition. If the British nurse wants her position improved she must not only speak, she must shout so loud that her voice will resound in the board-room and outside, so that the public and the committee will realise that her case demands attention.

"F. N." represents a class, and I want both her and others who agree with her views to see through my glasses. Without knowing it, and with the highest of motives, they are playing the game of those committees who would, according to "F. N.," threaten the persistent matron with dismissal. They are the enemies of progress. Indeed, I entertain no doubt that the most potent forces which operate against the profession are within rather than without the ranks. May I hark back to the Bedford-Fenwick opposition to the establishment of the College of Nursing, the only intelligent attempt at union that ever was made in this country? Sir Arthur Stanley was subjected to a storm of contumely because he was a layman, and yet he is the nearest approach that we have had to the knight in shining armour. And who made the clamour? The answer is—Nurses within the fold. Every conceivable effort to promote chaos and to prevent union that could be made has been and is being made, and it is not to be wondered at that the nurse, overworked and underpaid, is bewildered. Many of the advocates of disunion and agitation are dishonest, and are grinding their own axes. But there are others, and these I advise to climb to some high mountain and contemplate what is going on in the valley. We are in a new world, a world of reconstruction and reform. We are emerging from the throes of war, a war in which the nurse has played her part with a nobility and a self-sacrifice that is unsurpassable. In this new world the trained nurse may become one of the main factors of regeneration and reform. There are immense possibilities for the nurse, but if she remains in the background, and allows herself to be doped into silence and inactivity, she will miss the tide.

IERNE.

